# Stakeholder perspectives on short-stay joint replacement programs: results from a national cross-sectional study

**DOI:** 10.1186/s12913-023-10427-5

**Published:** 2023-12-18

**Authors:** Ilana N Ackerman, Danielle Berkovic, Sze-Ee Soh, Justine Naylor, Peter Lewis, Richard de Steiger, Rachelle Buchbinder, Zanfina Ademi, Patrick Vallance, Ian A Harris

**Affiliations:** 1 School of Public Health and Preventive Medicine, Monash University, Melbourne, Australia; 2 School of Primary and Allied Health Care, Monash University, Melbourne, Australia; 3 Liverpool Hospital, Liverpool, Australia; 4 School of Clinical Medicine, UNSW Medicine and Health, UNSW Sydney, Sydney, Australia; 5 Australian Orthopaedic Association National Joint Replacement Registry, Adelaide, Australia; 6 Faculty of Medicine, University of Adelaide, Adelaide, Australia; 7 Department of Surgery, Epworth HealthCare, University of Melbourne, Melbourne, Australia; 8 Health Economics and Policy Evaluation Research (HEPER) Group, Centre for Medicine Use and Safety, Faculty of Pharmacy and Pharmaceutical Sciences, Monash University, Melbourne, Australia; 9 Whitlam Orthopaedic Research Centre, Ingham Institute for Applied Medical Research, Sydney, Australia

**Keywords:** Enhanced recovery after surgery, Fast-track, Hip arthroplasty, Hip replacement, Knee arthroplasty, Knee replacement, Models of care, Short-stay joint replacement

## Abstract

**Background:**

The capacity to meet anticipated growth in joint replacement demand requires safe, efficient models of care. While short-stay joint replacement programs are being used internationally, they have not been widely implemented in many countries. Importantly, the critical challenges that need to be addressed ahead of large-scale program implementation remain unclear. This study aimed to investigate stakeholder perspectives on short-stay joint replacement programs, including perceived barriers and enablers to implementation and sustainability, and understand current practices in Australia.

**Methods:**

Four key stakeholder groups were invited to participate in this national study: (1) health professionals who provide joint replacement care; (2) hospital administrators involved in joint replacement provision; (3) patients with recent joint replacement; and (4) carers of people with recent joint replacement. Data on perceived feasibility (0 (not at all feasible) − 10 (highly feasible), appeal (0 (not at all appealing) − 10 (highly appealing), current practices, and barriers and enablers were collected using visual analogue scales, multiple response option and open-ended questions, via an online platform. Descriptive analysis and free-text content analysis was undertaken.

**Results:**

Data were available from 1,445 participants including 360 health professionals, 20 hospital administrators, 1,034 patients, and 31 carers. Short-stay program implementation was considered moderately feasible by health professionals (median 6, interquartile range (IQR) 3–8) and hospital administrators (median 5, IQR 5–6). Short-stay programs were moderately appealing to patients (median 7, IQR 2–9) but of little appeal to carers (median 3, IQR 1–7). Prominent implementation barriers included perceived limited appropriateness of short-stay programs, inadequate home supports, and issues around reimbursement models or program funding. Not having daily physiotherapy access and concerns about pain and mobility at home were common barriers for patients. Concern about patients’ ability to manage daily activities was the most common barrier for carers. Access to post-discharge services, better funding models, improved staffing, and consistent protocols and national care standards were prominent enablers.

**Conclusions:**

This national study has uniquely captured multiple stakeholder perspectives on short-stay joint replacement programs. The findings can guide future quality improvement and implementation initiatives and the development of resources to best support patients, carers, clinicians, and hospitals.

**Supplementary Information:**

The online version contains supplementary material available at 10.1186/s12913-023-10427-5.

## Background

Current rates of joint replacement surgery and forecast future demand necessitate efficient models of care that can safely shorten hospital admissions and maximise surgical throughput. Short-stay joint replacement programs (also known as ‘fast-track’, ‘rapid recovery’ or ‘enhanced recovery after surgery’ programs) are designed to reduce acute hospital length of stay through specific anaesthetic, analgesia, early mobilisation, and hospital discharge protocols [1]. Short-stay joint replacement programs have been introduced in numerous countries including the United States [2], United Kingdom [3], and Sweden [4], but do not feature prominently in the Australian health system. Consequently, acute hospital length of stay remains relatively long in Australia (average 3.5 days for hip replacement and 3.6 days for knee replacement in public hospitals [5]; average 4.3 days for hip replacement and 4.6 days for knee replacement in private hospitals [6]), compared to many other Organisation for Economic Co-operation and Development countries [7]. The use of inpatient rehabilitation after joint replacement remains unnecessarily high [8], given negligible value after uncomplicated procedures [9], and contributes to extended hospitalisation episodes. Over $AUD3.9 billion is spent annually on osteoarthritis care in Australia [10], with most health system expenditure attributed to joint replacement admissions. Significant opportunities exist for implementing contemporary models of care to support earlier patient discharge home, reduce low-value care, and minimise financial burden to the health system.

While high-quality evidence from Australian settings is not yet available, several quasi-experimental studies have reported a reduced hospital length of stay and encouraging patient outcomes after the implementation of short-stay joint replacement programs [11–14]. However, there are no national data on the use of these programs or quality considerations, including whether current programs align with international recommendations [15]. The acceptability and feasibility of short-stay joint replacement programs, and barriers and enablers to their implementation and sustainability, are not well understood. Identifying the potential concerns of patients and carers is also important for informing future implementation initiatives. Two recent studies, one involving a small sample of Australian private hospital patients [16], and the other involving general public perceptions of outpatient joint replacement in the United States [17], have provided preliminary insights but the views of other major stakeholders have not been collectively sought. Together, this information will enable consideration of the critical challenges and services that need to be developed and funded for larger-scale program implementation. This study aimed to investigate perceptions around short-stay joint replacement programs from health professional, hospital administrator, patient, and carer perspectives, and to understand current practices in Australia.

## Methods

### Study design

A national cross-sectional study.

### Participants

The following stakeholder groups were eligible to participate:

- Health professionals involved in the provision of hip or knee replacement care in Australia: specifically, orthopaedic surgeons, anaesthetists, general practitioners, nurses, and physiotherapists;
- Hospital administrators involved in the provision of hip or knee replacement services in Australia;
- People who had a hip or knee replacement in Australia within the last 12 months; and.
- Carers of people who had a hip or knee replacement in Australia within the last 12 months.

### Recruitment strategy

To enable time for stakeholder engagement, a multi-faceted strategy was developed to advertise the survey to potentially eligible individuals across Australia over a five-month period (6 September 2022 to 31 January 2023). Significant efforts were made to engage with peak professional bodies, community organisations, consumer advocacy groups, private health insurers, and academic clinician networks, with numerous organisations advertising the survey via their websites, member emails, newsletters, and/or social media. Snowballing methods were also used, with the survey advertisement being shared by individuals among their networks, including national anaesthetist special interest groups, practice nurse and orthopaedic nurse associations, and a national carer network, as well as via the researchers’ professional networks.

### Survey development and data collection

The survey content was developed by the multidisciplinary research team. Display logic was used to reduce responder burden and ensure that participants only saw items relevant to their stakeholder group and relevant to their previous responses. The survey was pilot tested to ensure functionality and to provide opportunity for questions and response options to be refined. The final survey included a mix of multiple response option questions, purpose-designed visual analogue scales, and open-ended questions.

Individuals who responded to the advertisement could contact the study team for further information or proceed directly to the online survey. The landing page provided an overview of the study and data storage principles. On the landing page, short-stay joint replacement programs were described as programs that ‘aim to shorten the hospital stay after hip or knee replacement surgery, with patients commonly going home from hospital 1–3 days after their surgery’. Outpatient or same-day discharge programs were not specifically mentioned. Individuals who chose to proceed were asked to identify the stakeholder group that best described them, before being directed to the relevant survey questions. As shown in Additional file 1, the questions were tailored to each stakeholder group but broadly covered basic demographics, feasibility, appeal, acceptability, and barriers and enablers. Short-stay joint replacement program details were captured in the health professional and hospital administrator questions. Where there was no current program, health professionals and hospital administrators were asked how feasible it would be to implement a short-stay program in the setting where they worked, on a scale from 0 (not at all feasible) to 10 (highly feasible). All health professional and hospital administrators were asked how acceptable these programs are (or would be), on a scale from 0 (not at all acceptable) to 10 (highly acceptable). Patients and carers were asked how appealing the idea of a short-stay joint replacement program would be, when considering a future hip or knee replacement, on a scale from 0 (not at all appealing) to 10 (highly appealing). Open-ended questions with free-text responses included questions regarding enablers to implementing and sustaining short-stay programs (for health professionals and hospital administrators), anything that would make the idea of a short-stay program more appealing (for patients and carers), anything needed to support them having a future hip or knee replacement as part of a short-stay program (for patients) or to support them to be a carer for someone having a hip or knee replacement as part of a short-stay program (for carers). All survey data collection was undertaken using the Qualtrics online platform and all responses were collected anonymously.

### Data analysis

Survey data were analysed descriptively using Stata 16/IC (StataCorp, Texas, USA), with separate analyses undertaken for each stakeholder group. Subgroup analyses were performed to understand the demographic characteristics of patients who did not perceive any barriers and to examine the appeal of short-stay programs for patients who lived alone versus those who did not (using chi-square tests or Mann-Whitney tests, as appropriate). Free-text responses were exported from Qualtrics into Excel (Microsoft Corporation) and analysed separately for each stakeholder group. These responses were analysed via content analysis, where each verbatim response was reviewed and designated a theme. The themes were not pre-specified by the researchers. The frequency of each theme was then examined, to identify the most common views for reporting. Where a verbatim response is cited, this is intended to provide an illustrative example of the relevant theme using the participant’s voice.

## Results

### Survey responses

There were 1,588 survey respondents. After removing respondents who did not progress beyond identifying their stakeholder group, data were available for analysis from 1,445 participants including 360 health professionals, 20 hospital administrators, 1,034 patients, and 31 carers.

### Participant characteristics

Characteristics of the health professional and hospital administrator participants are summarised in Table 1. Among the health professional participants, there were 193 (54%) physiotherapists, 64 (18%) orthopaedic surgeons, 56 (16%) anaesthetists, 43 (12%) nurses, and 4 (1%) general practitioners. Most were highly experienced, with 67% (n = 242) having ≥ 11 years’ experience in their profession and 20% (n = 73) having 6–10 years of experience. Most provided care for up to 10 patients undergoing hip or knee replacement each week (n = 253, 70%). Health professionals worked in public hospitals (n = 255, 71%), private hospitals (n = 149, 41%), and community-based settings (n = 37, 10%), with some working in multiple settings. Health professionals worked in metropolitan (n = 267, 74%), regional (n = 83, 23%), and rural areas (n = 19, 5%), including combinations of these. Hospital administrators worked in private hospital settings (n = 12, 60%), public hospitals (n = 7, 35%), and other settings including insurance (n = 2, 10%), with one working in public and private hospitals.

**Table 1:** Characteristics of health professional and hospital administrator participants

Characteristic	Health professionals (n = 360)	Hospital administrators (n = 20)
Profession, n (%)		
Orthopaedic surgeon	64 (18)	N/A
Anaesthetist	56 (16)	N/A
General practitioner	4 (1)	N/A
Nurse	43 (12)	N/A
Physiotherapist	193 (54)	N/A
Years of practice*, n (%)		
<1 year	3 (< 1)	2 (10)
1–5 years	42 (12)	7 (35)
6–10 years	73 (20)	5 (25)
11 + years	242 (67)	6 (30)
Geographical location, n (%)		
Metropolitan area	267 (74)	16 (80)
Regional area	83 (23)	4 (20)
Rural area	19 (5)	0 (0)
Setting type, n (%)		
Public hospital	255 (71)	7 (35)
Private hospital	149 (41)	12 (60)
Community health centre	11 (3)	N/A
Community-based practice or private practice	23 (6)	N/A
Aged care facility	3 (< 1)	N/A
Other (including insurance)	10 (3)	2 (10)
Number of patients each week, n (%)		
<5 patients	137 (38)	N/A
5–10 patients	116 (32)	N/A
11–20 patients	47 (13)	N/A
>20 patients	47 (13)	N/A

N/A: not applicable for this stakeholder group

Percentages may exceed 100% where participants could select more than one option

*Years in current role for health administrator participants

The patient and carer participant characteristics are presented in Table 2. The majority were aged ≥ 60 years (n = 895, 86%) and women (n = 613, 59%). One-quarter of patients lived alone (n = 270, 26%). Forty-five per cent had received a hip replacement in the past year (n = 467); 56% (n = 578) had received a knee replacement. Most procedures were undertaken in private hospital settings (97% for hip replacement; 98% for knee replacement). Most carers were aged ≥ 50 years (n = 26, 84%) and most were women (n = 28, 90%).

**Table 2:** Characteristics of patient and carer participants

Characteristic	Patients (n = 1,034)	Carers (n = 31)
Age, n (%)		
20–29 years	0 (0)	1 (3)
30–39 years	0 (0)	1 (3)
40–49 years	18 (2)	3 (10)
50–59 years	121 (12)	9 (29)
60–69 years	374 (36)	8 (26)
70 + years	521 (50)	9 (29)
Gender, n (%)		
Woman	613 (59)	28 (90)
Man	418 (40)	2 (6)
Non-binary / gender-diverse	0 (0)	0 (0)
I use a different term or prefer not to say	3 (< 1)	1 (3)
Lives alone, n (%)		
Yes	270 (26)	N/A
No	764 (74)	N/A
Hip replacement in past year*, n (%)	467 (45)	17 (55)
Hip replacement setting, n (%)		
Public hospital	15 (3)	6 (35)
Private hospital	451 (97)	10 (59)
Missing or unsure	1 (< 1)	1 (6)
Knee replacement in past year*, n (%)	578 (56)	18 (58)
Knee replacement setting, n (%)		
Public hospital	11 (2)	4 (22)
Private hospital	566 (98)	14 (78)

N/A: not applicable for this stakeholder group

*Provided care for someone who had a hip or knee replacement in the past year for carer participants

### Awareness of short-stay programs

Most health professionals (n = 322, 89%) and hospital administrators (n = 19, 95%) reported awareness of the short-stay joint replacement concept. Lower awareness was evident among patients (n = 564, 55% were aware of this concept) and carers (n = 11, 35% were aware).

### Perceived feasibility of implementing a short-stay program

Health professionals and hospital administrators considered that implementing a short-stay program would be moderately feasible in the setting where they worked (median 6, IQR 3–8 and median 5, IQR 5–6, respectively).

### Perceived acceptability and appeal of short-stay programs

Among health professionals, the perceived acceptability of short-stay programs to patients was high (median score 8, IQR 6–9). Hospital administrators perceived these programs to be highly acceptable to hospital management (median 8, IQR 6–10), and moderately acceptable to health professionals (median 6, IQR 5–9) and patients (median 7, IQR 5–8).

Overall, short-stay programs were moderately appealing to patients (median 7, IQR 2–9). However, the programs were of less appeal to those who lived alone (median 5 versus 7 for patients who did not live alone, p = 0.01 for Mann-Whitney test). Patients expressed that home-based pain management of the same quality as in hospital, increased allied health support at home (particularly physiotherapy), support to undertake activities of daily living (for example, food preparation and showering, for those who lived alone), better co-ordination of care at home, knowledge that infection risk was lower at home, and reduced costs would make short-stay programs more appealing to them. Patients reported that pain management, increased allied health and home supports, better co-ordination of care, support for partners, rebates as an incentive to leave hospital earlier, and enhanced efforts to instill confidence were needed to support them having a future hip or knee replacement as part of a short-stay program.

On average, short-stay programs were of little appeal to carers (median 3, IQR 1–7). Carers indicated that better pain management for patients in their care, patient access to a medical team and at-home care for several days, accurate assessment of the patient’s mobility and ability to complete activities of daily living, knowing that the patient was ready to be at home, and education about situations where the patient should present to an emergency department would make short-stay programs more appealing to them. Lower risk of hospital-acquired infections and reduced travel time (to/from the hospital) were also perceived as enablers by carers. Provision of information (including wound care information), advice and support around helping with activities of daily living and mobility, allied health support at home, access to the medical team, monitoring for hospital-acquired infection, and better communication around expectations were cited as examples of what carers would need to support them.

### Current Australian practices in short-stay joint replacement

Forty-six per cent of health professionals (n = 167) reported there was currently a short-stay joint replacement program where they worked, or that they currently provided care within a short-stay program. The components of these programs are summarised in Table 3. Nine of the 20 hospital administrators (45%) reported a current short-stay joint replacement program in their hospital. The program components aligned closely with those reported by health professionals, with most programs including pre-operative patient education (n = 8, 89%), early mobilisation (n = 7, 78%), home-based support (n = 7, 78%), and standardised discharge criteria (n = 5, 56%).

**Table 3:** Components of current short-stay programs, as reported by health professionals

Characteristic	n = 167
Early mobilisation within 24 h of surgery, n (%)	152 (91)
Pre-operative patient education, n (%)	140 (84)
Multi-modal anaesthesia, n (%)	114 (68)
Standardised discharge criteria, n (%)	112 (67)
Anti-thrombosis prophylaxis protocol, n (%)	111 (66)
Use of local anaesthesia, n (%)	101 (60)
Home-based support, n (%)	101 (60)
Oral analgesia, n (%)	99 (59)
Anti-microbial prophylaxis protocol, n (%)	83 (50)
Standardised anaesthetic protocol, n (%)	77 (46)
Nausea or vomiting prophylaxis protocol, n (%)	72 (43)
Fluid management, n (%)	67 (40)
Blood conservation measures, n (%)	54 (32)
Early nutrition, n (%)	51 (31)
Liberal pre-operative fasting regimen, n (%)	23 (14)
Unsure, n (%)	17 (10)
Other*, n (%)	7 (5)

*Includes pre-operative physiotherapy, ‘clean surgery’, daily review by acute pain service, follow-up phone call from physiotherapist or perioperative nurse, app-based follow-up by surgeon’s clinic, and team management

### Experiences with short-stay programs

Most of the 167 health professionals involved with short-stay programs reported a positive experience with providing this care (‘strongly positive’: n = 74, 44%; ‘somewhat positive’: n = 68, 41%). Six health professionals (4%) reported a ‘neutral’ experience while negative experiences (‘somewhat negative’ or ‘strongly negative’) were only reported by 4 participants (2%). Fifteen health professionals (9%) did not respond to this question.

In total, 246 patients reported they received their recent joint replacement as part of a short-stay program. When asked about their experience, most reported it was positive (‘strongly positive’: n = 160, 65%; ‘somewhat positive’: n = 46, 19%). Seven per cent reported a ‘neutral’ experience (n = 18) while few reported negative experiences (n = 12, 5% for ‘somewhat negative’ and ‘strongly negative’ combined). Four per cent (n = 10) did not respond to this question.

### Perceived barriers to implementing short-stay programs

**Fig. 1:**
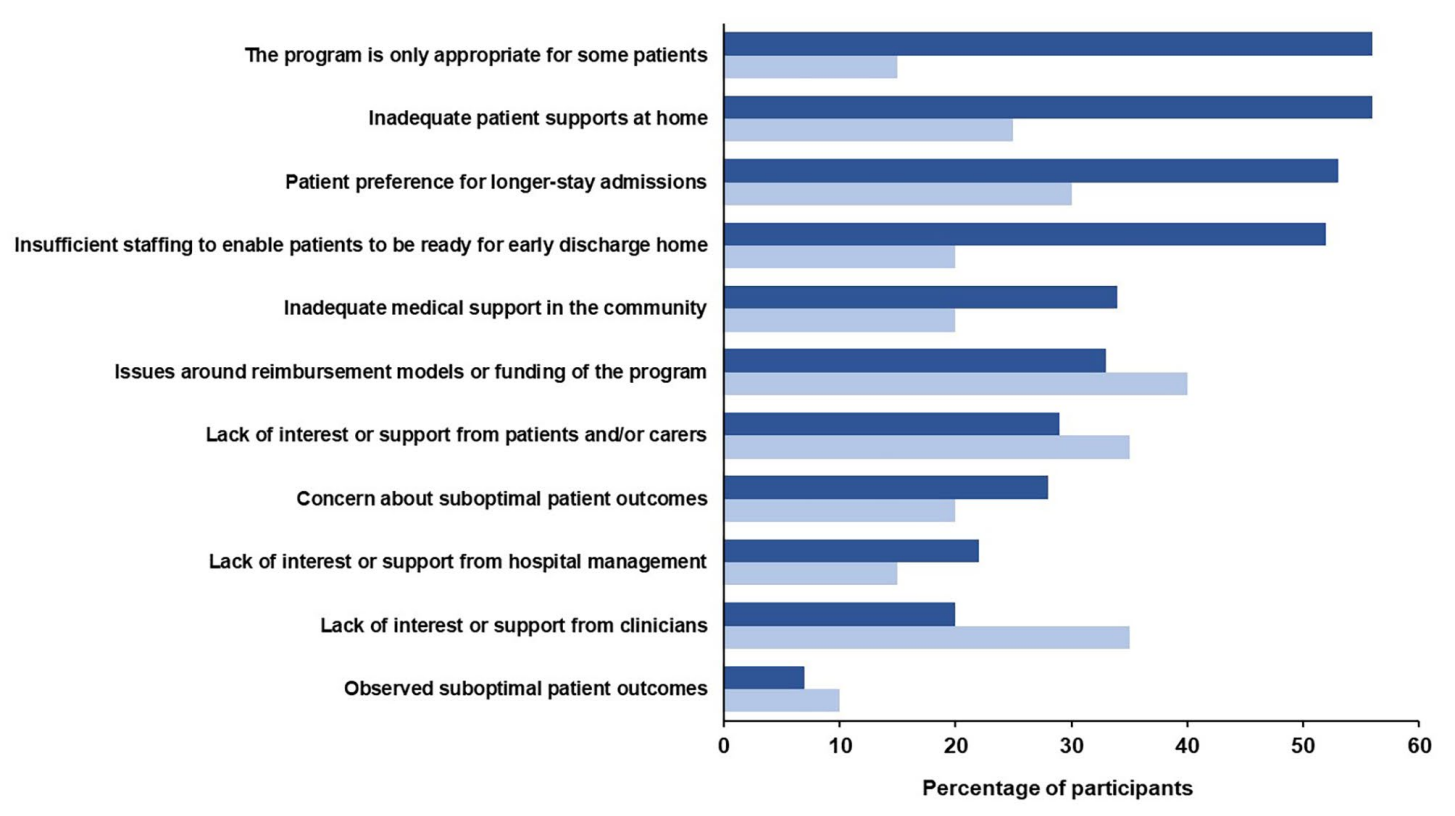
Perceived barriers to implementing a short-stay program. Dark blue bars represent health professional responses (n = 360) and light blue bars represent hospital administrator responses (n = 20)

Health professionals and hospital administrators perceived a range of barriers to implementing short-stay joint replacement programs in the setting where they worked (Fig. 1). For health professionals, these were most frequently around the limited appropriateness of these programs (n = 202, 56%), inadequate home supports for patients (n = 200, 56%), patient preference for longer-stay admissions (n = 189, 53%), and insufficient staffing to ensure patients were ready for early discharge (n = 188, 52%). For hospital administrators, reimbursement models or funding issues were the most frequently perceived barrier, followed by a lack of interest or support from clinicians, patients and/or carers (Fig. 1).

### Factors perceived to affect the sustainability of short-stay programs

Inadequate patient supports at home (n = 183, 51%) and insufficient staffing to enable patient readiness for early discharge (n = 179, 50%) were the most frequently cited barriers to sustainability in Australia, as perceived by health professionals (Additional file 1, Figure A1). Consistent with the barriers to implementation, hospital administrators perceived that issues around reimbursement models or program funding, and lack of interest or support from clinicians, patients and/or carers were the greatest barriers to sustainability (Additional file 1, Figure A1).

### Barriers to joint replacement as part of a short-stay program, as perceived by patients and carers

**Fig. 2:**
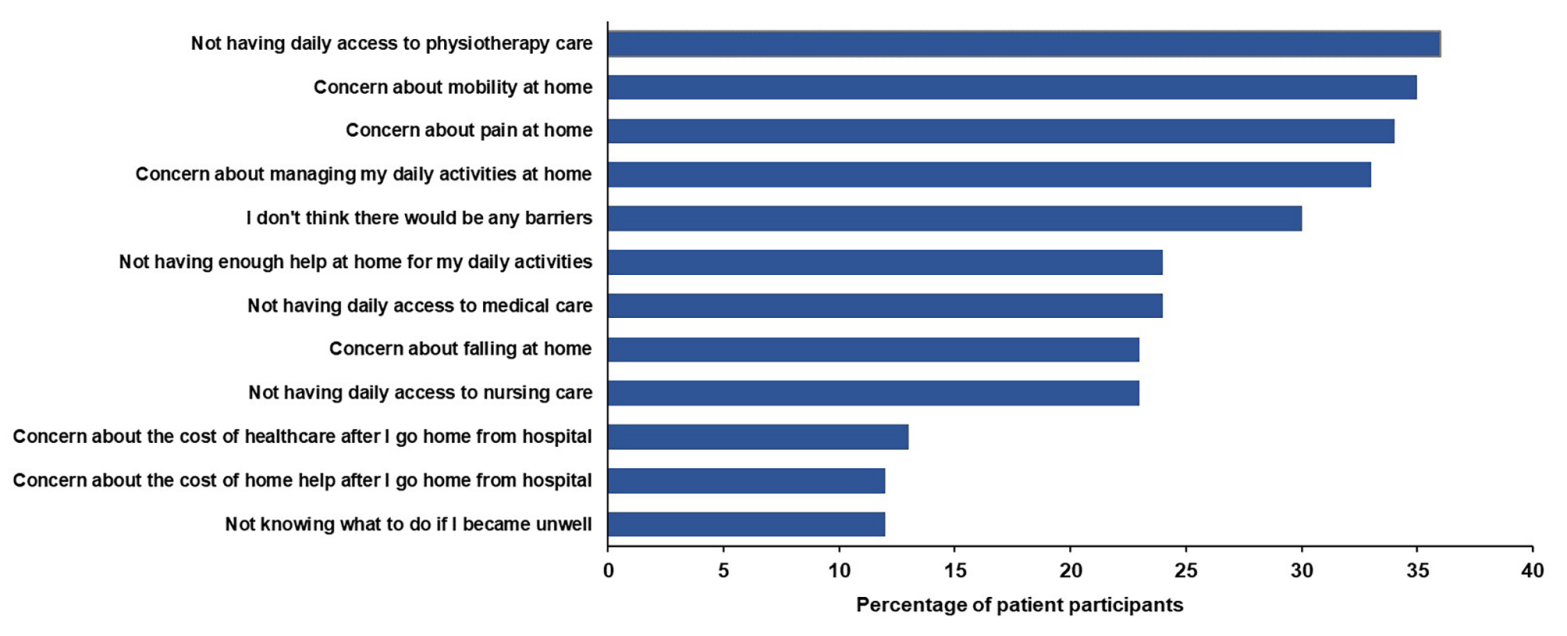
Patient-perceived barriers to having future joint replacement as part of a short-stay program (n = 1,034)

For patients, not having daily access to physiotherapy care and concern about their mobility, pain and managing daily activities at home were the most frequently identified barriers to having future joint replacement as part of a short-stay program (Fig. 2). Not having enough help at home, not having daily access to medical or nursing care, and concerns about falling at home were also commonly reported. However, 30% of patient participants (n = 306) did not perceive any barriers; this subgroup had a similar age distribution (chi-square = 0.98, p = 0.81) but included a higher proportion of men (chi-square = 45.64, p < 0.01) and a lower proportion of individuals living alone (chi-square = 20.10, p < 0.01), compared to participants who reported barriers or were unsure. Carers were most frequently concerned about the patient’s ability to manage at home (n = 19, 61%), the patient’s requirement for more help with daily activities (n = 17, 55%), the patient’s pain (n = 15, 48%) or mobility at home (n = 15, 48%), and that the patient may fall at home (n = 14, 45%). Not having daily access to medical, nursing, and physiotherapy care (n = 13, 42% for each) were also commonly perceived as barriers by carers. Some carers were also concerned they could not provide the help needed (n = 9, 29%) and that they would not know what to do if the patient became unwell (n = 8, 26%).

### Enablers as perceived by health professionals

Four key enablers were identified by health professionals, with regard to implementing short-stay joint replacement programs in Australia:

1. Greater accessibility to services and supports: this included routine access to allied health pre-operative assessment and education, community-based rehabilitation services, more supports in the home, adequate home services, better pain management, fast-stream rehabilitation, home-based interventions, improved community supports with shorter waitlists, smooth referral to outpatient rehabilitation, community-based medical options, and access to home nursing.
2. Changes to funding approaches: this included better funding models for the private sector, funding for the public sector to trial fast-track models, funding for staff to cover day of surgery mobilisation and on weekends, removal of private health insurance requirements for a minimum length of hospital stay, funding of post-operative rehabilitation in private practices, funding for allied health staff to attend pre-admission clinics and manage patient expectations, funding for allied health staff to support patients at home, financial disincentives for longer hospital lengths of stay, investment in community-based services, and more funding for people living in rural and remote communities.
3. Better education and understanding: this included education to patients and families to address expectations for the hospital stay and plan for a shorter stay, surgeon education regarding home rehabilitation, a better understanding of short-stay models from health funds, hospital, medical, nursing and allied health, national education programs targeted at public education, clear expectations by all members of the team before surgery, consideration around language (for example, using the term ‘enhanced recovery’) and messaging (for example, re-framing elective surgery as a procedure that is planned and prepared for, *“…the replacement procedure happens in the hospital, the recovery happens at home”*).
4. Improved staffing: this included staffing to support the intensity of therapy required, flexible working arrangements for staff (including evening and weekend staffing), enhanced allied health staffing, dedicated staffing for short-stay programs, and the need for appropriate workforce models.

Health professionals also cited the need for broad professional support for short-stay programs; for example, from hospital management, the relevant professional colleges, ‘buy in’ from medical and surgical staff, and support from general practitioners. The need for formal frameworks and documented guidance was also raised. Suggestions for this included national standards, guidelines, clear and consistent protocols, and clinical pathways that could support appropriate patient selection and program governance.

Similar themes were identified with respect to sustaining short-stay joint replacement programs. Greater accessibility of services (particularly community-based healthcare and support services), appropriate funding models, and addressing staffing issues (around inpatient and community-based care) were perceived as key enablers. Having consistent, standardised protocols across health services, having national standards or guidelines for joint replacement care for low complexity patients, and having clear pathways that include pre-operative planning were also identified as key enablers.

### Enablers as perceived by hospital administrators

Hospital administrators cited improvements in the evidence base *(“gold standard research demonstrating improved clinical, patient experience, and business outcomes”*), collaborative care between clinicians, hospitals, and health insurers, education (around patient expectations and value, community education to improve patient understanding, and education for visiting medical officers), and funding considerations *(“clear and fair funding from health funds”*, and *“supportive funding models”*) as the key enablers for implementing short-stay programs in Australia. Improved community resources were also identified as a key enabler, as illustrated by the response “*At present, I work in an area with a very diverse population, with patients of diverse backgrounds. We do not have the community supports or the community programs to facilitate early return home for a majority of these patients*.”

When considering sustainability, hospital administrators cited a favourable cost-benefit analysis, favourable patient outcomes and safety profile, adequate resources, continuing education, and funding models (for example, longer-term contractual arrangements) as key enablers. Having sufficient resources was also a key theme; this included having appropriate policies and procedures, adequate patient ‘prehabilitation’ and follow-up, out-of-hospital care models to meet patient needs, home-based, outpatient, and community-based rehabilitation options, and home supports for activities of daily living and transport.

## Discussion

Incorporating the views of 1,445 health professionals, hospital administrators, patients, and carers, this national study has uniquely investigated perceptions around short-stay joint replacement programs, including factors perceived to impact implementation and sustainability. By capturing diverse stakeholder perspectives, this research has generated detailed insights to inform future national implementation strategies and enable due consideration of common concerns. While a range of views were expressed, there was overlap in the perceived enablers to implementation and sustainability; namely, the need for post-discharge supports; education and resources to support health professionals, patients and carers; and attention to appropriate funding models. Focusing on shared opportunities to address issues that are common to several stakeholder groups will likely be the most efficient path towards national implementation.

To date, our understanding of the use of short-stay joint replacement programs in Australia has been restricted to non-randomised or single group research studies [11–14] and a pilot evaluation in the private healthcare sector [18]. Our national data indicate that short-stay programs are being used in Australian hospital settings and that existing program components largely align with Enhanced Recovery After Surgery (ERAS®) Society recommendations [15]. The most commonly used components were early mobilisation and pre-operative education, although other strongly recommended elements (for example, early nutrition, limited pre-operative fasting, and blood conservation measures [15]) were less frequently used. A common implementation barrier for health professionals was the limited appropriateness of short-stay programs. Anecdotal reports suggest patients with few co-morbidities, at lower risk of complications, and with home supports are most likely to be selected for short-stay joint replacement programs. As cited by health professional participants in our study, there is a need for clear and consistent protocols, clinical pathways and/or national guidelines to support appropriate patient selection and perioperative care. However, the evidence around optimal patient selection for short-stay joint replacement is limited, and patient selection criteria are not mentioned within current recommendations [15]. Generating high-quality evidence to guide patient selection (with consideration of demographic characteristics, clinical factors, and home-based supports) should be a focus of future short-stay research.

This study has provided valuable insights into patient and carer perspectives. We found that short-stay programs were moderately appealing to patients (less so for those who lived alone), although awareness was limited. Two other studies have considered patient perspectives around short-stay programs [16, 19] and our research demonstrated similar enabling factors, including the desire for support to undertake activities of daily living, better co-ordination of care at home, and greater support for carers. A recent ‘crowd-sourcing’ survey in the United States examined the general public’s preferences and perceptions around outpatient joint replacement, but did not specifically include people with personal experience of surgery [17]. The concerns around mobility and pain management reported by patients and carers in our survey align closely with a recent qualitative evidence synthesis focusing on factors influencing the implementation of early discharge and hospital-at-home programs more broadly [20]. The paucity of carer-focused research around short-stay joint replacement programs is highlighted by a recent scoping review that sought to examine the impact of outpatient joint replacement on informal caregivers at home [21]. The review found no published studies on carer burden, despite the vital support they provide to patients following early hospital discharge. We are only aware of a small Danish qualitative study (10 participants) that explored spouses’ experiences of a case management intervention provided as part of a fast-track hip replacement program [22]. The impact of early discharge models of care (not limited to joint replacement) on carers and the lack of formal recognition and accessible information for them has been highlighted in a recent qualitative evidence synthesis [20]. In our study, carers did not find short-stay programs appealing and had little awareness of these programs. Our data also demonstrate that the anticipated physical and emotional burden on carers following early hospital discharge was a concern for carers as well as patients. Together, these data indicate a need for appropriate pre-operative patient and carer education (including around the concept of short-stay joint replacement and potential benefits) and highlight the importance of providing clear information upon discharge including mechanisms for contacting the hospital or care providers should issues arise.

This study has several strengths. It has concurrently examined the perspectives of multiple stakeholder groups with high relevance to joint replacement care. Although mindful of responder burden, we designed the survey to cover a breadth of concepts that could inform future implementation activities. We advertised the survey nationally through major professional, health, and consumer organisations and a large private health insurer, to capture diverse experiences across Australia’s parallel public and private healthcare systems. We also acknowledge the study limitations, including the potential for responder bias. Recruitment of general practitioner participants was limited and we recognise that other clinicians who may be involved in joint replacement care (for example, occupational therapists and rehabilitation physicians) were not included. As the majority of patient participants received surgery in the private sector, we recognise that the perspectives of public patients may be under-represented, although insights were provided by health professional and hospital administrator participants who worked in public hospital settings. For context, we note that the majority of primary joint replacement procedures in Australia are performed in private hospitals [23]. Finally, the survey did not ask about potential downstream outcomes after short-stay program implementation (for example, cost savings, changes to resource requirements, changes in surgical volume, or additional hospital bed capacity for other patient groups).

## Conclusions

This national study has provided a new understanding of health professional, hospital administrator, patient and carer perspectives around short-stay joint replacement programs, and a snapshot of contemporary programs in Australia. The findings around awareness, acceptability, and perceived barriers and enablers can assist with planning future implementation initiatives, including consideration of the essential supports nominated by each stakeholder group.

### Electronic supplementary material

Below is the link to the electronic supplementary material.

Additional file 1: National stakeholder survey. Figure A1. Perceived barriers to the sustainability of short-stay joint replacement programs

## Data Availability

The stakeholder survey is available in Additional file 1. The survey data are not publicly available under current ethics approvals but the analysis code may be obtained from the authors upon request.

## Abbreviations

ERAS: Enhanced Recovery After Surgery
IQR: Interquartile range
