# Supplementary material for: Stakeholder perspectives on short-stay joint replacement programs: results from a national cross-sectional study
Source: BMC Health Serv Res. 2023 Dec 18;23:1436. doi: 10.1186/s12913-023-10427-5 (PMC10729344; doi:10.1186/s12913-023-10427-5)
Supplement: Supplementary file 1 — Additional file 1: National stakeholder survey. Figure A1. Perceived barriers to the sustainability of short-stay joint replacement programs [file 12913_2023_10427_MOESM1_ESM.docx]

**Stakeholder perspectives on short-stay joint replacement programs: Results from a national cross-sectional study**

Ilana N Ackerman, Danielle Berkovic, Sze-Ee Soh, Justine Naylor, Peter Lewis, Richard de Steiger, Rachelle Buchbinder, Zanfina Ademi, Patrick Vallance, Ian A Harris

**Additional file 1**

**Survey used for the national stakeholder study**

Please note: Display logic / skip functionality (not shown in this document) was used to ensure that participants were only shown questions that were relevant to them, based on their stakeholder group and responses to previous questions

**Landing page (seen by all participants)**

**Short-stay joint replacement survey**

Welcome and thank you for your interest in this survey. Our multidisciplinary team, led by researchers at Monash University, is undertaking a national stakeholder survey on short-stay joint replacement programs (also known as ‘fast track’ or ‘enhanced recovery’ programs). **Short-stay programs aim to shorten the hospital stay after hip or knee replacement surgery, with patients commonly going home from hospital 1-3 days after their surgery.**

We are inviting four groups of participants to take part in this survey:

- Health professionals in any setting who currently provide care to people undergoing hip or knee replacement surgery - this includes orthopaedic surgeons, anaesthetists, general practitioners, nurses, and physiotherapists;
- Hospital administrators in any setting who are currently involved in the provision of hip or knee replacement services;
- People who have had a hip or knee replacement within the past 12 months; and
- Carers of people who have had a hip or knee replacement within the past 12 months.

The survey takes less than 10 minutes to complete. The questions are designed to help us understand the types of short-stay joint replacement programs that are currently in place as well as programs that have been used previously. We are also interested in understanding your thoughts on these programs and factors that might affect the use of short-stay programs in Australia. **You can still take part in the survey even if you do not have any personal experience of short-stay joint replacement programs.**

This research is funded by an Innovation Grant awarded by the (details removed for blinding). Ethics approval for this research has been obtained from (details removed for blinding).

**All survey responses are anonymous** and all data will be stored on a secure online platform at Monash University. Only the Monash researchers will have access to the data. All data relating to this study will be deleted 5 years after any publication of the results, in accordance with Monash University guidelines.

On behalf of the research team, thank you again for your time and interest. If you would like any further information about this survey, please contact (names and contact details removed).

**Please click on the arrow below to start the survey**

NB - please ensure that you complete all survey questions in one sitting - as the survey is anonymous, it is not possible to exit and return to the questions at a later time

**Please select the option below that best describes you (seen by all participants)**

- I am an orthopaedic surgeon, anaesthetist, general practitioner, nurse or physiotherapist who currently provides care to people undergoing hip or knee replacement surgery in Australia
- I am a hospital administrator who is currently involved in the provision of hip or knee replacement services in Australia
- I have received a hip or knee replacement in Australia within the past 12 months
- I am a carer for someone who has received a hip or knee replacement in Australia within the past 12 months

**Demographics questions for health professional participants**

- Are you…?
- An orthopaedic surgeon
- An anaesthetist
- A general practitioner
- A nurse
- A physiotherapist
- For how many years (full-time equivalent) have you practised in your profession?
- <1 year
- 1-5 years
- 6-10 years
- 11 years or more
- In which Australian state or territory do you currently work?
- Australian Capital Territory
- New South Wales
- Northern Territory
- Queensland
- South Australia
- Tasmania
- Victoria
- Western Australia
- Do you currently work in…? (please select all that apply)
- A metropolitan area
- A regional area
- A rural area
- In what setting(s) do you currently work? (please select all that apply)
- Public hospital
- Private hospital
- Community health centre
- Community-based practice or private practice
- Aged care facility
- Other
- If ‘other’, please specify the type of setting you currently work in (free text)
- Approximately how many patients undergoing hip or knee replacement surgery do you provide care to each week (either before, during and/or after surgery)?
- Less than 5 patients
- Between 5-10 patients
- Between 11-20 patients
- More than 20 patients

**Proceed to short-stay program questions for health professional participants**

**Demographics questions for hospital administrator participants**

- For how many years (full-time equivalent) have you been in your current role?
- <1 year
- 1-5 years
- 6-10 years
- 11 years or more
- In which Australian state or territory do you work?
- Australian Capital Territory
- New South Wales
- Northern Territory
- Queensland
- South Australia
- Tasmania
- Victoria
- Western Australia
- Do you currently work in…? (please select all that apply)
- A metropolitan area
- A regional area
- A rural area
- In what setting(s) do you currently work? (please select all that apply)
- Public hospital
- Private hospital
- Other
- If ‘other’, please specify the type of setting you currently work in

**Proceed to short-stay program questions for hospital administrator participants**

**Demographics questions for patient participants**

- What is your age?
- <20 years
- 20-29 years
- 30-39 years
- 40-49 years
- 50-59 years
- 60-69 years
- 70 years or over
- Do you identify as?
- Woman
- Man
- Non-binary / gender diverse
- I use a different term
- Prefer not to say
- In which Australian state or territory do you live?
- Australian Capital Territory
- New South Wales
- Northern Territory
- Queensland
- South Australia
- Tasmania
- Victoria
- Western Australia
- Do you live alone?
- Yes
- No
- Have you had a hip replacement in the past year?
- Yes
- No
- Did you receive this hip replacement in a…?
- Public hospital
- Private hospital
- Have you had a knee replacement in the past year?
- Yes
- No
- Did you receive this knee replacement in a…?
- Public hospital
- Private hospital

**Proceed to short-stay program questions for patient participants**

**Demographics questions for carer participants**

- What is your age?
- <20 years
- 20-29 years
- 30-39 years
- 40-49 years
- 50-59 years
- 60-69 years
- 70 years or over
- Do you identify as?
- Woman
- Man
- Non-binary / gender diverse
- I use a different term
- Prefer not to say
- In which Australian state or territory do you live?
- Australian Capital Territory
- New South Wales
- Northern Territory
- Queensland
- South Australia
- Tasmania
- Victoria
- Western Australia
- Have you provided care for someone who had a hip replacement in the past year?
- Yes
- No
- Did this person receive their hip replacement in a…?
- Public hospital
- Private hospital
- Unsure
- Have you provided care for someone who had a knee replacement in the past year?
- Yes
- No
- Did you receive this person receive their knee replacement in a…?
- Public hospital
- Private hospital
- Unsure

**Proceed to short-stay program questions for carer participants**

**Short-stay program questions for health professional participants**

- Are you aware of the concept of short-stay joint replacement programs (programs which aim to shorten the hospital stay, with patients commonly going home from hospital 1-3 days after their surgery)?
- Yes
- No
- Is there currently a short-stay joint replacement program in the setting(s) where you work, or do you currently provide care to patients who receive joint replacement as part of a short-stay program?
- Yes
- No
- Unsure
- How feasible do you consider it would be to implement a short-stay joint replacement program in the setting(s) where you work?
- Sliding scale from 0 (not at all feasible) to 10 (highly feasible)
- Has there previously been a short-stay joint replacement program in the setting(s) where you work?
- Yes
- No
- Unsure
- For how long was this short-stay joint replacement program in place? (free text response)
- What was the length of stay target for this short-stay program?
- 1 day (same-day surgery and discharge from hospital)
- 2 days (an overnight stay)
- 3 days
- 4 days
- More than 4 days
- Unsure
- What components were included in this short-stay program? (please select all that apply)
- Pre-operative patient education
- Liberal pre-operative fasting regimen
- Standardised anaesthetic protocol
- Use of local anaesthesia
- Multi-modal analgesia
- Oral analgesia
- Blood conservation measures
- Fluid management
- Anti-thrombosis prophylaxis protocol
- Anti-microbial prophylaxis protocol
- Nausea or vomiting prophylaxis protocol
- Early nutrition
- Early mobilisation (within 24 hours of surgery)
- Standardised discharge criteria
- Home-based support (eg. nursing and/or physiotherapy)
- Other
- Unsure
- If other, please specify the other components of this short-stay program (free text response)
- What is the main reason(s) why this short-stay program is no longer in place?
- Lack of interest or support from hospital management
- Lack of interest or support from clinicians
- Lack of interest or support from patients and/or carers
- Issues around reimbursement models or funding of the program
- Insufficient staffing to enable patients to be ready for early discharge home
- Observed suboptimal patient outcomes
- Concern about suboptimal patient outcomes
- Patient preference for longer-stay admissions
- The program was only appropriate for some patients
- Inadequate patient supports at home
- Inadequate medical support in the community
- Other
- Unsure
- If other, please specify the other reason for why this short-stay program is no longer in place (free text response)
- What components are included in this short-stay program (select all that apply)?
- Pre-operative patient education
- Liberal pre-operative fasting regimen
- Standardised anaesthetic protocol
- Use of local anaesthesia
- Multimodal analgesia
- Oral analgesia
- Blood conservation measures
- Fluid management
- Anti-thrombosis prophylaxis protocol
- Anti-microbial prophylaxis protocol
- Nausea or vomiting prophylaxis protocol
- Early nutrition
- Early mobilisation (within 24 hours of surgery)
- Standardised discharge criteria
- Home-based support (eg. nursing and/or physiotherapy)
- Other
- Unsure
- If other, please specify the other components of the short-stay program (free text response)
- How would you describe your experience with providing care to patients who undergo hip or knee replacement as part of a short-stay program?
- Strongly positive
- Somewhat positive
- Neutral
- Somewhat negative
- Strongly negative
- Please explain the reason(s) for this response (free text response)
- How acceptable do you think short-stay joint replacement programs are (or would be) to patients who are undergoing hip or knee replacement?
- Sliding scale from 0 (not at all acceptable) to 10 (highly acceptable)
- What do you perceive to be barriers to implementing short-stay joint replacement programs in the setting(s) where you work? (please select all that apply)
- Lack of interest or support from hospital management
- Lack of interest or support from clinicians
- Lack of interest or support from patients and/or carers
- Issues around reimbursement models or funding of the program
- Insufficient staffing to enable patients to be ready for early discharge home
- Observed suboptimal patient outcomes
- Concern about suboptimal patient outcomes
- Patient preference for longer-stay admissions
- The program is only appropriate for some patients
- Inadequate patient supports at home
- Inadequate medical support in the community
- Other
- Unsure
- If other, please specify any other barriers to implementation (free text response)
- What factors do you consider might affect the sustainability of short-stay joint replacement programs in Australia? (please select all that apply)
- Lack of interest or support from hospital management
- Lack of interest or support from clinicians
- Lack of interest or support from patients and/or carers
- Issues around reimbursement models or funding of the program
- Insufficient staffing to enable patients to be ready for early discharge home
- Observed suboptimal patient outcomes
- Concern about suboptimal patient outcomes
- Patient preference for longer-stay admissions
- The program is only appropriate for some patients
- Inadequate patient supports at home
- Inadequate medical support in the community
- Other
- Unsure
- If other, please specify any other barriers to sustainability (free text response)
- What would make it easier to implement short-stay joint replacement programs in Australia? (free text response)
- What would make it easier to sustain short-stay joint replacement programs in Australia? (free text response)
- In your opinion, what further evidence is needed before short-stay joint replacement programs can be more broadly implemented in Australia? (free text response)

**End of health professional questions (proceed to final page)**

**Short-stay program questions for hospital administrator participants**

- Are you aware of the concept of short-stay joint replacement programs (programs which aim to shorten the hospital stay, with patients commonly going home from hospital 1-3 days after their surgery)?
- Yes
- No
- Is there currently a short-stay joint replacement program in the hospital(s) where you work?
- Yes
- No
- Unsure
- How feasible do you consider it would be to implement a short-stay joint replacement program in the hospital(s) where you work?
- Sliding scale from 0 (not at all feasible) to 10 (highly feasible)
- Has there previously been a short-stay joint replacement program in the hospital(s) where you work?
- Yes
- No
- Unsure
- For how long was this short-stay joint replacement program in place? (free text response)
- What was the length of stay target for the short-stay program?
- 1 day (same-day surgery and discharge from hospital)
- 2 days (an overnight stay)
- 3 days
- 4 days
- More than 4 days
- Unsure
- What components were included in this short-stay program? (please select all that apply)
- Pre-operative patient education
- Liberal pre-operative fasting regimen
- Standardised anaesthetic protocol
- Use of local anaesthesia
- Multimodal analgesia
- Oral analgesia
- Blood conservation measures
- Fluid management
- Anti-thrombosis prophylaxis protocol
- Anti-microbial prophylaxis protocol
- Nausea or vomiting prophylaxis protocol
- Early nutrition
- Early mobilisation (within 24 hours of surgery)
- Standardised discharge criteria
- Home-based support (eg. nursing and/or physiotherapy)
- Other
- Unsure
- If other, please specify these other components of the short-stay program (free text response)
- What is the main reason(s) why this short-stay program is no longer in place?
- Lack of interest or support from hospital management
- Lack of interest or support from clinicians
- Lack of interest or support from patients and/or carers
- Issues around reimbursement models or funding of the program
- Insufficient staffing to enable patients to be ready for early discharge home
- Observed suboptimal patient outcomes
- Concern about suboptimal patient outcomes
- Patient preference for longer-stay admissions
- The program was only appropriate for some patients
- Inadequate patient supports at home
- Inadequate medical support in the community
- Other
- Unsure
- If other, please specify the other reason(s) for why this short-stay program is no longer in place (free text response)
- What components are included in this program (please select all that apply)?
- Pre-operative patient education
- Liberal pre-operative fasting regimen
- Standardised anaesthetic protocol
- Use of local anaesthesia
- Multimodal analgesia
- Oral analgesia
- Blood conservation measures
- Fluid management
- Anti-thrombosis prophylaxis protocol
- Anti-microbial prophylaxis protocol
- Nausea or vomiting prophylaxis protocol
- Early nutrition
- Early mobilisation (within 24 hours of surgery)
- Standardised discharge criteria
- Home-based support (eg. nursing and/or physiotherapy)
- Other
- Unsure
- If other, please specify the other components of the short-stay program (free text response)
- How acceptable do you think short-stay joint replacement programs are (or would be) to hospital management?
- Sliding scale from 0 (not at all acceptable) to 10 (highly acceptable)
- How acceptable do you think short-stay joint replacement programs are (or would be) to health professionals who provide care to patients undergoing hip or knee replacement?
- Sliding scale from 0 (not at all acceptable) to 10 (highly acceptable)
- How acceptable do you think short-stay joint replacement programs are (or would be) to patients who are undergoing hip or knee replacement?
- Sliding scale from 0 (not at all acceptable) to 10 (highly acceptable)
- What do you perceive to be the main barriers to implementing short-stay joint replacement programs in the hospital(s) where you work? (please select all that apply)
- Lack of interest or support from hospital management
- Lack of interest or support from clinicians
- Lack of interest or support from patients and/or carers
- Issues around reimbursement models or funding of the program
- Insufficient staffing to enable patients to be ready for early discharge home
- Observed suboptimal patient outcomes
- Concern about suboptimal patient outcomes
- Patient preference for longer-stay admissions
- The program is only appropriate for some patients
- Inadequate patient supports at home
- Inadequate medical support in the community
- Other
- Unsure
- If other, please specify any other barriers to implementation (free text response)
- What factors do you consider might affect the sustainability of short-stay joint replacement programs in Australia? (please select all that apply)
- Lack of interest or support from hospital management
- Lack of interest or support from clinicians
- Lack of interest or support from patients and/or carers
- Issues around reimbursement models or funding of the program
- Insufficient staffing to enable patients to be ready for early discharge home
- Observed suboptimal patient outcomes
- Concern about suboptimal patient outcomes
- Patient preference for longer-stay admissions
- The program is only appropriate for some patients
- Inadequate patient supports at home
- Inadequate medical support in the community
- Other
- Unsure
- If other, please specify any other barriers to sustainability (free text response)
- What would make it easier to implement short-stay joint replacement programs in Australia? (free text response)
- What would be needed in order to sustain a short-stay joint replacement program in Australia? (free text response)
- In your opinion, what further evidence is needed before short-stay joint replacement programs can be more broadly implemented in Australia? (free text response)

**End of hospital administrator questions (proceed to final page)**

**Short-stay program questions for patient participants**

- Are you aware of the concept of short-stay joint replacement programs (programs which aim to shorten the hospital stay, with patients commonly going home from hospital 1-3 days after their surgery)?
- Yes
- No
- Did you receive your recent hip or knee replacement as part of a short-stay joint replacement program?
- Yes
- No
- Unsure
- How would you describe your experience with receiving your recent hip or knee replacement as part of a short-stay program?
- Strongly positive
- Somewhat positive
- Neutral
- Somewhat negative
- Strongly negative
- Please explain the reason(s) for this response (for example, why you may have had a positive or negative experience) (free text response)
- How many days did you stay in the acute hospital (where you had your operation) after your recent hip or knee replacement?
- 1 day (same-day surgery and discharge from hospital)
- 2 days (an overnight stay)
- 3 days
- 4 days
- More than 4 days
- Unsure
- When considering a future hip or knee replacement, how appealing would the idea of a short-stay joint replacement program be to you?
- Sliding scale from 0 (not at all appealing) to 10 (highly appealing)
- Is there anything that would make the idea of a short-stay joint replacement program more appealing to you? (free text response)
- What would be the main barriers for you, in having a future hip or knee replacement provided as part of a short-stay program? (please select all that apply)
- I don’t think there would be any barriers
- Concern about managing my daily activities at home
- Concern about pain at home
- Concern about mobility at home
- Concern about falling at home
- Concern about the cost of healthcare after I go home from hospital
- Concern about the cost of home help after I go home from hospital
- Not having enough help at home for my daily activities
- Not knowing what to do if I become unwell
- Not having daily access to medical care
- Not having daily access to nursing care
- Not having daily access to physiotherapy care
- Other
- Unsure
- If other, please specify any other concerns you may have (free text response)
- Is there anything you would need to support you to have a future hip or knee replacement as part of a short-stay joint program? (free text response)

**End of patient questions (proceed to final page)**

**Short-stay program questions for carer participants**

- Are you aware of the concept of short-stay joint replacement programs (programs which aim to shorten the hospital stay, with patients commonly going home from hospital 1-3 days after their surgery)?
- Yes
- No
- Have you provided care for someone who received their recent hip or knee replacement as part of a short-stay joint replacement program?
- Yes
- No
- Unsure
- As a carer, how appealing is the idea of a short-stay joint replacement program to you?
- Sliding scale from 0 (not at all appealing) to 10 (highly appealing)
- As a carer, is there anything that would make the idea of a short-stay joint replacement program more appealing to you? (free text response)
- As a carer, what would be the main barriers for you with regards to a short-stay joint replacement program? (please select all that apply)
- I don’t think there would be any barriers
- Concern about the patient’s ability to manage their daily activities at home
- Concern about the patient’s pain at home
- Concern about the patient’s mobility at home
- Concern that the patient may fall at home
- Concern about the patient needing more help at home with their daily activities
- Concern about the cost of healthcare after the patient goes home from hospital
- Concern about the cost of home help after the patient goes home from hospital
- Not knowing what to do if the patient becomes unwell
- Not having daily access to medical care
- Not having daily access to nursing care
- Not having daily access to physiotherapy care
- Concern that I could not provide the help they may need
- Other
- Unsure
- If other, please specify any other concerns you may have (free text response)
- Is there anything you would need to support you to be a carer for someone who has a hip or knee replacement as part of a short-stay joint program? (free text response)

**End of carer questions (proceed to final page)**

**Final page (seen by all participants)**

**Short-stay joint replacement survey**

Your survey responses have been recorded and you may now close this window.

Thank you for taking the time to participate in this national survey.

**
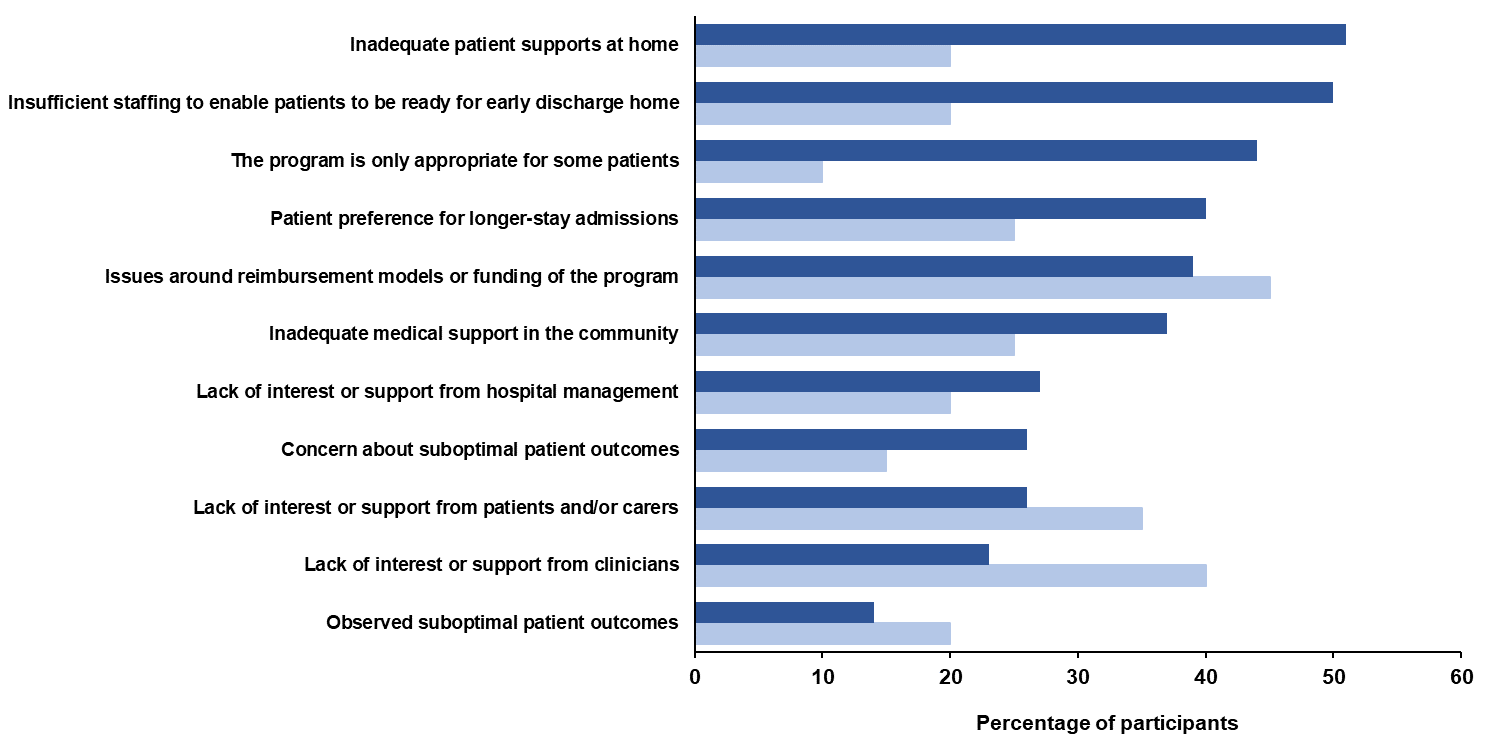
**

**Figure A1. Perceived barriers to the sustainability of short-stay joint replacement programs**

Dark blue bars represent health professional responses (n=360) and light blue bars represent hospital administrator responses (n=20)
